# Radial Head Antibiotic Spacer for the Treatment of Prosthetic Joint Infection Following a Radial Head Arthroplasty: A Case Report

**DOI:** 10.7759/cureus.77276

**Published:** 2025-01-11

**Authors:** Daniel Calem, David Ahn, Nicholas Genovese, Irfan Ahmed, Michael Vosbikian

**Affiliations:** 1 Orthopaedic Surgery, Rutgers University New Jersey Medical School, Newark, USA; 2 Orthopaedic Surgery, Beth Israel Deaconess Medical Center, Harvard Medical School, Boston, USA

**Keywords:** 2-stage revision, antibiotic spacer, arthroplasty, infection control measures, radial head

## Abstract

Prosthetic joint infection (PJI) following radial head arthroplasty (RHA) is a rare, yet devastating complication. Current treatment options for PJI comprise debridement, antibiotics, and implant retention (DAIR); resection arthroplasty; and one- or two-stage revision. Two-stage revision is the current gold standard for the treatment of PJI at the hip, shoulder, and knee, and involves using an antibiotic spacer to maintain limb length and soft tissue tension. Despite the widespread use of this technique in the treatment of PJI at other sites, its use in the treatment of RHA PJI has not been described. In this report, we illustrate the use of a novel polymethylmethacrylate (PMMA) spacer construct for the treatment of RHA PJI by the two-stage revision in a male patient. This method shows promising results, with the spacer maintaining structural integrity for four months.

## Introduction

Prosthetic joint infection (PJI) following radial head arthroplasty (RHA) is a rare, yet devastating complication, occurring in up to 4% of cases [1]. Managing radial head PJI remains can be challenging as there are no standard guidelines on treatment. Treatment modality is generally based on the treating surgeon’s experience and evidence derived from studies of PJIs following elbow, shoulder, hip, and knee replacement [2,3]. For cases of PJI following total elbow arthroplasty specifically, two-stage revision arthroplasty is associated with a lower rate of recurrent infection [4,5]. The available treatment options for RHA PJI include debridement, antibiotics, and implant retention (DAIR); resection arthroplasty; and one- or two-stage revision [6].

Treatment of PJI is further complicated if the primary RHA is performed in the setting of trauma, particularly if the patient has sustained an unstable elbow injury such as a medial collateral ligament (MCL) injury or Essex-Lopresti lesion. In such scenarios, resection arthroplasty would be contraindicated, as the radial head prosthesis is critical in maintaining axial stability of the forearm and acts as a secondary stabilizer to valgus stress at the elbow [7]. Hence, resection arthroplasty is considered a salvage procedure and is reserved for RHA PJI without any concomitant instability, longitudinal forearm deficiency, and distal radioulnar joint (DRUJ) injury. When there is loosening of the prosthesis and there remains sufficient bone stock available for reimplantation, a revision arthroplasty is indicated.

The use of an antibiotic-loaded cement spacer in combination with targeted antibiotic therapy has been well described for two-staged revisions for hip, shoulder, and knee PJIs [2,3]. The same strategy has been described for periprosthetic elbow infections [8,9], but there is no published data on infection control and functional outcomes following radial head PJI. The use of an antibiotic cement spacer has many benefits, including local delivery of antibiotics to the infected joint space, preservation of elbow motion, maintenance of soft tissue tension, stabilization to valgus stress, and providing axial stability to the forearm in cases of longitudinal forearm deficiency. A study by Capomassi et al. showed satisfactory results using a polymethylmethacrylate (PMMA) spacer to temporarily rebuild an unstable elbow after radial head fracture in circumstances where a definitive radial head prosthesis is not available [10]. Complications following the use of cement spacers are primarily mechanical in nature, with fracture and dislocation being the most common.

To our knowledge, no study of the use of an antibiotic-loaded cement spacer with a metal screw endoskeleton for the treatment of RHA PJI has been published so far. This report aims to describe a simple and reproducible technique for creating an antibiotic-loaded cement radial head spacer for the treatment of RHA PJI.

## Case presentation

Case

**Figure 1:**
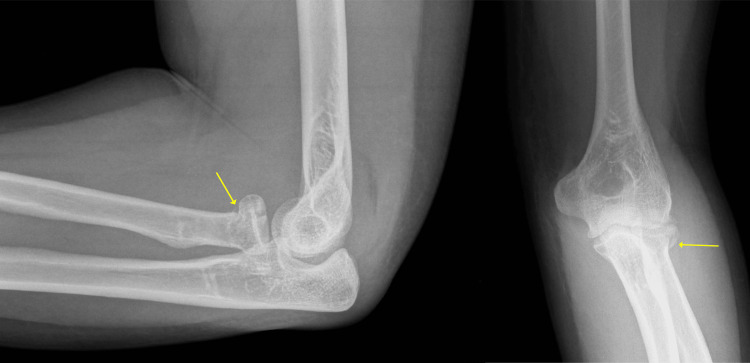
Post-injury X-rays The lateral and anteroposterior elbow views of the post-injury film are shown in the left and right panels, respectively. The yellow arrows demonstrate the impacted radial head fracture

**Figure 2:**
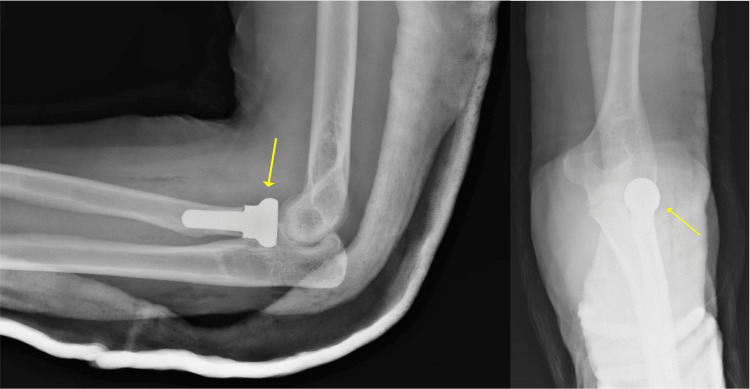
Post-radial head arthroplasty X-rays The lateral and anteroposterior views of the elbow after initial radial head arthroplasty are shown in the left and right panels, respectively. The yellow arrows demonstrate the radial head prosthesis implantation with adequate alignment

The patient was a 50-year-old, HIV-positive, right-hand dominant male who presented to the emergency department with left elbow pain after being struck by a car two weeks prior. He had been initially evaluated at an outside hospital and told that there were no fractures or dislocations at the time of the accident. On presentation, he had swelling at the left elbow with tenderness to palpation at the radial head. The passive range of motion (ROM) at the left elbow was 10-100 degrees of flexion; he had full ROM otherwise and was neurovascularly intact. Imaging showed a mildly displaced left radial head fracture, subluxation of the elbow joint, and a coronoid fracture (Figure 1). The patient was taken to the OR for his terrible triad injury and underwent left RHA, lateral ulnar collateral ligament (LUCL) repair, and open reduction and internal fixation (ORIF) of the coronoid (Figure 2).

**Figure 3:**
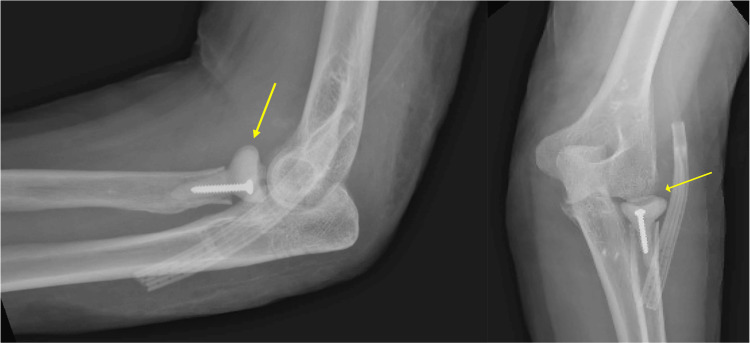
Post-radial head cement spacer placement X-rays The lateral and anteroposterior views of the elbow after the radial head cement spacer placement are shown in the left and right panels, respectively. The yellow arrows demonstrate an antibiotic radial head spacer molded over a 3.5 x 22 mm cortical screw, articulating with the lateral humeral condyle

A month after the surgery, the patient returned to the ED with complaints of fevers, chills, and pain. The physical exam revealed a punctate opening in the dorsolateral aspect of the left elbow with active thin, white, non-purulent discharge. A CT scan with intravenous contrast demonstrated a rim-enhancing fluid collection in the soft tissue overlying the radial head prosthesis. The patient was diagnosed with RHA PJI and was taken back to the OR for irrigation and debridement (I&D). The previous radial head hardware was explanted, and an antibiotic spacer was constructed over a 3.5 x 22 mm cortical screw using PALACOS MV+G bone cement (Heraeus Medical GmbH, Wehrheim, Germany), which contains 0.5 g of gentamycin per 40 g bag, with an additional 1 g of vancomycin per 40 g bag incorporated. The spacer was sized to match the diameter of the 24 mm implant but made 2-4 mm shorter as the spacer was a monoblock (Figure 3). Cultures were sent for microbiologic analysis, which later grew Streptococcus pyogenes. On repeat washout two days later, antibiotic beads with 0.5 g vancomycin and 1 g tobramycin were constructed and placed. The patient was discharged home with a peripherally inserted central catheter and a prescription for home antibiotics.

Due to noncompliance with home antibiotics, the patient required multiple I&Ds in the subsequent months. Over this period, the patient was found to have a superinfection with methicillin-resistant staph aureus (MRSA), requiring alteration of the antibiotic regimen. Additionally, the radial head spacer was exchanged. The spacer was maintained for four months for infection control and until ligamentous structures healed. Due to continued antibiotic noncompliance and subsequent osteomyelitis, the patient ultimately underwent spacer removal and proximal radius saucerization.

**Figure 4:**
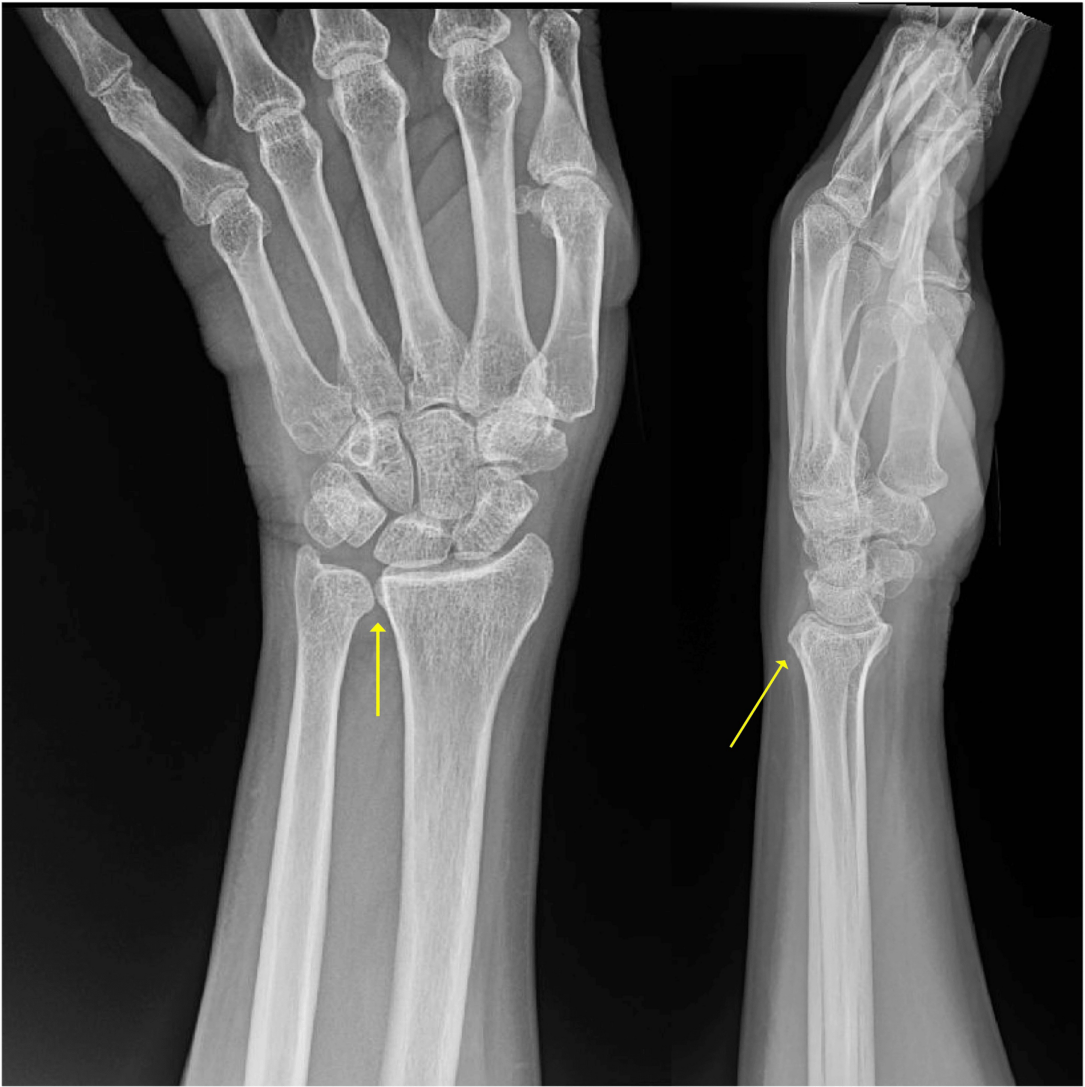
Final follow-up wrist X-rays The anteroposterior and lateral views of the wrist at the final follow-up are shown in the left and right panels, respectively. On the anteroposterior view, the yellow arrow indicates that there is no widening at the distal radioulnar joint (DRUJ), with adequate alignment. On the lateral view, the yellow arrow indicates adequate alignment between the radius and ulna, without any radiographic signs of volar or dorsal ulnar subluxation

**Figure 5:**
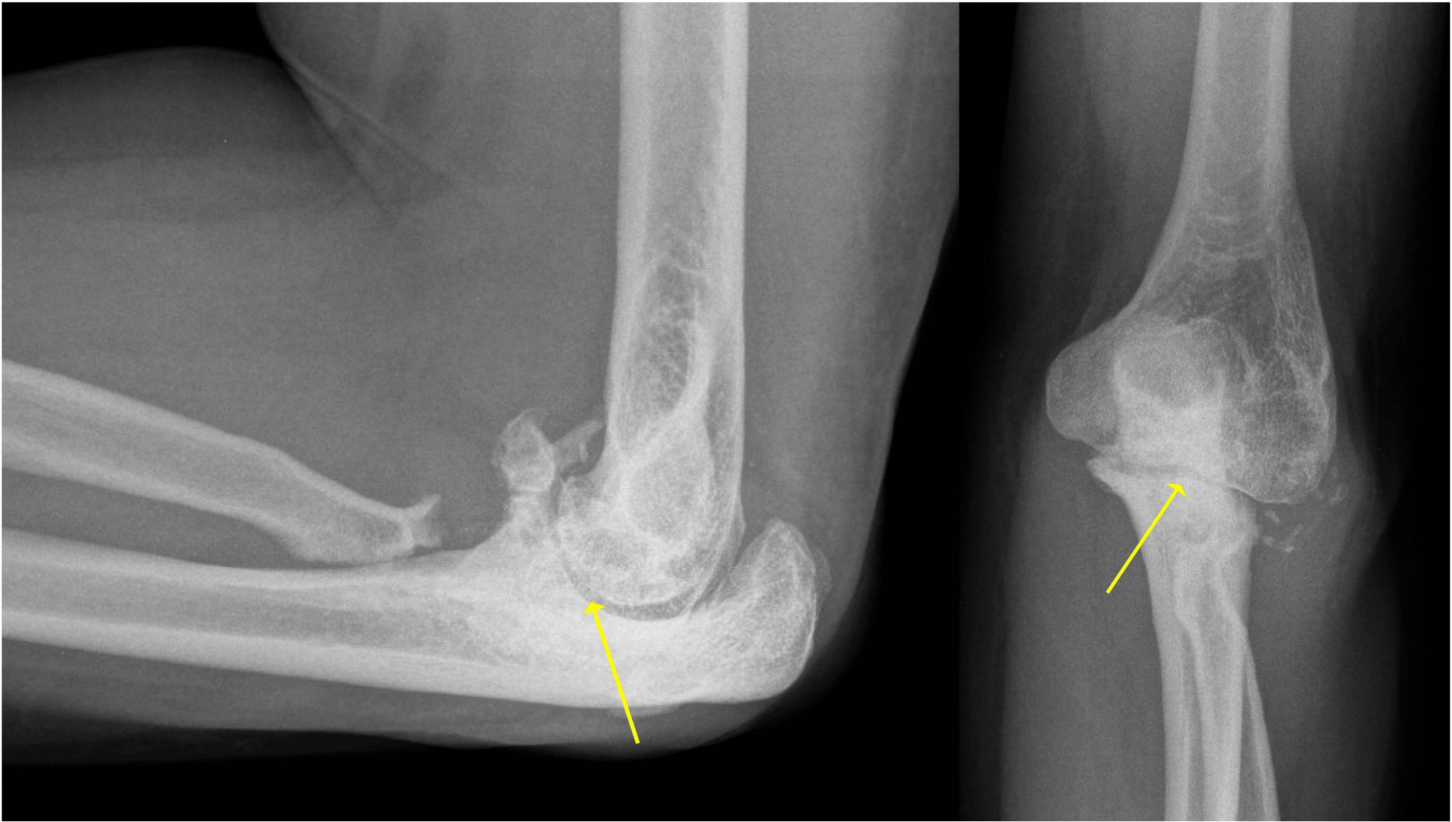
Final follow-up elbow X-rays The lateral and AP anteroposterior views of the elbow at the final follow-up are shown in the left and right panels, respectively. The yellow arrows demonstrate joint space narrowing at the ulnar-trochlear joint

The final follow-up was conducted six years after the index placement of the radial head spacer. Physical exam showed maintenance of ROM since prior surgeries, with 15 degrees short of full extension at the elbow. He had full ROM in flexion and pronosupination. There was no DRUJ instability (Figure 4) or coronal instability at the elbow. Labs showed no signs of infection. Radiographs obtained showed severe arthritic changes at the elbow (Figure 5). However, the patient was content with his outcome and denied further management at that time.

Technique

The patient is placed supine on the operating table with the operative extremity placed on a radiolucent hand table. A non-sterile brachial tourniquet is utilized. Antibiotic administration should be held until tissue cultures have been obtained to increase bacterial yield in the absence of systemic toxicity or sepsis.

The previous lateral surgical incision is opened, and subcutaneous suprafascial planes are developed keeping the fat with the skin to respect vascularity. For a radial head prosthetic joint infection that has a draining wound, the wound should first be debrided by excision of the wound margins and extended until there is healthy bleeding tissue. The prior deep surgical interval is identified via the suture line at the level of the joint, and an arthrotomy is performed. In the setting of prior ligamentous repair, the lateral collateral ligamentous complex should be identified and protected. A sample of fluid and tissue from the joint should be sent for anaerobic, aerobic, fungal, and mycobacterial cultures, as well as gram stain.

Once specimens have been obtained, perioperative antibiotics may be administered. Next, all infected material must be thoroughly removed including the prosthesis, cement, and any surrounding granulation tissue. In the setting of a well-fixed cemented prosthesis, osteotomes may be used to aid in the removal. After thorough debridement has been performed, the surrounding tissue and proximal radius should be thoroughly irrigated with sterile saline. Our preferred method involves utilizing a laparoscopic irrigator or cystoscopy tubing and 9 liters of 0.9% sterile saline to irrigate the joint and surrounding tissues.

**Figure 6:**
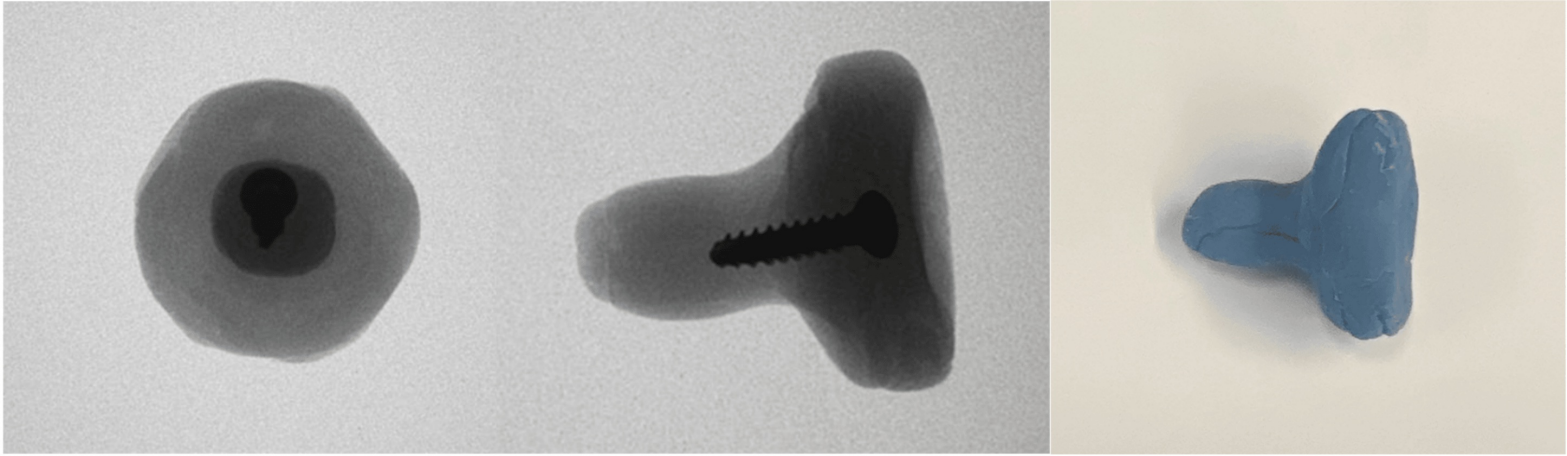
Antibiotic-loaded cement radial head spacer The left and middle panels show a radiographic image of the antibiotic cement spacer molded over a 3.5 x 22 mm cortical screw. The right panel shows a gross image of the antibiotic cement spacer, which was matched in size to the previously explanted radial head prosthesis

The explanted radial head prosthesis is then used as a template to construct an appropriately sized radial head antibiotic-loaded cement spacer. Using PALACOS MV+G cement (Heraeus Medical GmbH), which contains 0.5 g of gentamycin per 40 g bag, an additional 1 g of vancomycin is incorporated into the cement and a radial head spacer is constructed over a Synthes 3.5 x 22 mm cortical screw (Figure 6). As the radial head-neck junction is susceptible to fracture, this screw serves as an endoskeleton and acts as a rebar, improving the mechanical properties of the spacer. The spacer should be sized to match the previously explanted radial head prosthesis. It is recommended to shorten the spacer by 2-4 mm given that this technique utilizes monoblock rather than a modular prosthesis. Once hardened and cooled, the cement spacer is inserted into the proximal radius. Fluoroscopic images should be obtained to ensure appropriate spacer position and congruity of the joint. Meticulous hemostasis should be obtained to minimize the risk of postoperative hematoma. The wound is again irrigated, and the lateral structures are closed with 0-PDS sutures. A drain is then placed in the subcutaneous tissues. The skin is closed with 3-0 nylons in a horizontal mattress configuration.

Postoperatively, the elbow is temporarily immobilized in a posterior long arm splint until the incision is healed and sutures are removed, generally 14 days postoperatively. Broad-spectrum intravenous antibiotics are continued until positive joint cultures are reported, then tailored appropriately for pathogen-specific antibiotic therapy. The drain is left in for 48 hours postoperatively or until drain output is less than 30 cc/24-hour period. Once sutures are removed and the incision is healed, patients are encouraged to begin the range of motion of the elbow. Early implementation of elbow motion therapy exercises is crucial for postoperative recovery.

Infectious disease consultation should be made in the immediate postoperative period to help guide the management and treatment of the infection. Once inflammatory markers stabilize, an antibiotic holiday is recommended to evaluate the efficacy of the treatment. If the infection settles, a second-stage reimplantation can then be performed.

## Discussion

When managing PJI following RHA, it is imperative to have a thorough understanding of the pertinent anatomy and the important function the radial head has in stabilizing the elbow. The radial head articulates with both the capitellum and proximal ulna. Anatomical studies have demonstrated that the head has an ellipsoid and conical shape with variable offset [11]. The radial side of the radiocapitellar joint is convex and covered by articular cartilage. It is a secondary restraint to valgus force and is responsible for roughly a third of the support in this direction [12-14]. In addition, the radial head has been shown to act as a constraint to posterolateral, axial, and varus loading [15,16].

Following the disruption of the MCL, the radial head is the main stabilizer against valgus and compressive forces [13,17]. The radial head also provides axial stability to the forearm in concert with the interosseous membrane and distal radioulnar joint. In the presence of an MCL injury or an Essex-Lopresti lesion, radial head excision further exacerbates valgus and axial instability respectively, and can lead to proximal radial migration. Therefore, performing a radial head arthroplasty will help restore normal radiocapitellar contact, which is critical to the coronal and longitudinal stability of the elbow and forearm [16,18].

A patient who presents with a history of prior radial head replacement complaining of elbow pain, decreased elbow function, and/or clinical signs of infection should be evaluated for a radial head prosthetic joint infection. While there are no universally accepted guidelines for the diagnosis of RHA PJI, the British Elbow and Shoulder Society recommends utilizing the Musculoskeletal Infection Society (MSIS) criteria for the diagnosis of periprosthetic elbow infections [19]. The MSIS criteria for diagnosing a PJI are as follows: a sinus tract communicating with the prosthesis, a pathogen present in at least two tissue or fluid samples from the prosthetic joint, and/or a combination of multiple elevated inflammatory markers [20].

If infection is suspected, inflammatory markers should be analyzed, including erythrocyte sedimentation rate (ESR) and C-reactive protein (CRP). X-rays should be obtained to assess loosening, malalignment, subluxation, or dissociation of the prosthesis. CT scans are useful to evaluate any surrounding abscesses or sinus tracts communicating with the joint that will need to be excised. When RHA PJI is suspected but diagnostic uncertainty exists, joint aspiration may provide additional knowledge for antibiotic selection until definitive information regarding the joint state is known. However, in cases of obvious infection, such as when one major MSIS criteria is present, we prefer urgent surgical intervention rather than performing aspiration first.

The indications for this procedure include an infected radial head prosthesis in patients with reasonable bone stock and/or in the setting of associated ligamentous instability. The antibiotics used in the cement spacer involve a combination of gentamycin and vancomycin; therefore, it is important to assess for any allergies or sensitivities a patient may have to the antibiotics utilized for this technique. Furthermore, clinicians should be mindful of the patient's comorbidities, which may impede recovery from multiple surgical procedures.

To our knowledge, this is the first documented case in the literature about the use of an antibiotic radial head spacer for the management of RHA PJI. Our patient’s treatment was complicated by his immunosuppressed status, antibiotic noncompliance, recurrent infection, and eventual osteomyelitis. While the patient required multiple surgical I&Ds, the spacer provided an excellent temporary solution in treating the radial head PJI while also maintaining joint stability given the initial concomitant ligamentous injury. The spacer was maintained for four months until ligamentous structures were healed, and elbow stability was restored. The patient eventually required resection arthroplasty 10 months following the index terrible triad injury due to osteomyelitis and remains on long-term suppressive oral antibiotics.

Recurrent infection may occur and therefore it is crucial to ensure complete debridement of all necrotic and foreign material. A multidisciplinary approach with aggressive surgical intervention and prolonged combination antimicrobial therapy is essential for a successful outcome. Patients with radial head prosthetic joint infections also suffer from complications of septic destruction of the joint, which will typically result in arthritis, leading to pain, loss of motion, and functional disability. Repeated open exposure of the joint adds to potential iatrogenic complications including damage to the articular cartilage, potential injury of the posterior interosseous nerve, heterotopic ossification, and/or damage to the collateral ligaments.

Complications relating to improper sizing of the spacer also need to be considered. Oversizing will typically generate excess friction of the capitellum leading to chondromalacia, pain, and loss of motion. In contrast, an undersized spacer may result in valgus or posterolateral instability. Furthermore, there is also the risk of osteolysis of the proximal radius metaphysis caused by excess movement of the stalk of the spacer inside the medullary cavity. In severe cases, this could cause the stalk of the spacer to break, which can be potentially avoided by the use of a screw inside the spacer as described in our technique, which functions as a rebar to improve the structural integrity of the spacer.

Infection after a radial head arthroplasty is a difficult challenge to manage. Options include lifelong antibiotic suppression, resection arthroplasty, or one or two-stage revision. Resection arthroplasty is reserved for patients who have no associated ligamentous instability. While revision procedures have been shown to successfully eradicate the infection in total elbow arthroplasty PJI, there are no published data regarding infection control and functional outcomes following radial head PJI.

## Conclusions

While the antibiotic-loaded radial head cement spacer is a monoblock construct, it still permits functional use of the elbow. The benefits of utilizing an articulating radial head spacer include the preservation of tissue length and tension and limited muscle atrophy, thereby optimizing function if a subsequent two-stage surgery is performed. Early initiation of elbow motion is key for reducing edema, inflammation, joint fibrosis, and contracture. It provides a mechanical force for fluid circulation, which offers the potential for the removal of proinflammatory mediators. We propose that the above-described method is safe and effective and should be considered in select patients.
